# Behavioral Periodicity Detection from 24 h Wrist Accelerometry and Associations with Cardiometabolic Risk and Health-Related Quality of Life

**DOI:** 10.1155/2016/4856506

**Published:** 2016-01-31

**Authors:** Matthew P. Buman, Feiyan Hu, Eamonn Newman, Alan F. Smeaton, Dana R. Epstein

**Affiliations:** ^1^Arizona State University, Phoenix, AZ 85004, USA; ^2^Dublin City University, Dublin, Ireland; ^3^Phoenix Veterans Affairs Health Care System, Phoenix, AZ 85012, USA

## Abstract

Periodicities (repeating patterns) are observed in many human behaviors. Their strength may capture untapped patterns that incorporate sleep, sedentary, and active behaviors into a single metric indicative of better health. We present a framework to detect periodicities from longitudinal wrist-worn accelerometry data. GENEActiv accelerometer data were collected from 20 participants (17 men, 3 women, aged 35–65) continuously for 64.4 ± 26.2 (range: 13.9 to 102.0) consecutive days. Cardiometabolic risk biomarkers and health-related quality of life metrics were assessed at baseline. Periodograms were constructed to determine patterns emergent from the accelerometer data. Periodicity strength was calculated using circular autocorrelations for time-lagged windows. The most notable periodicity was at 24 h, indicating a circadian rest-activity cycle; however, its strength varied significantly across participants. Periodicity strength was most consistently associated with LDL-cholesterol (*r*'s = 0.40–0.79, *P*'s < 0.05) and triglycerides (*r*'s = 0.68–0.86, *P*'s < 0.05) but also associated with hs-CRP and health-related quality of life, even after adjusting for demographics and self-rated physical activity and insomnia symptoms. Our framework demonstrates a new method for characterizing behavior patterns longitudinally which captures relationships between 24 h accelerometry data and health outcomes.

## 1. Introduction

Human behaviors that are measured by accelerometer—sleep, sedentary behavior, and more active behaviors—are consistently associated with cardiometabolic risk biomarkers and health-related quality of life [1–4]. Recently, substitution-based and compositional models have been used to better characterize the combined or joint impact these behaviors may have on health [5–8]. Accelerometers can also capture the patterns in which sleep, sedentary, and active behaviors are accumulated. For example, physical activity accumulated in bouts of ≥10 min have stronger relationships with health outcomes than total physical activity [9]. Discontinuous sedentary time is less detrimental for health than sedentary time accumulated in continuous bouts [10]. Finally, in sleep, accelerometers can quantify measures of sleep quality (e.g., sleep efficiency, wake after sleep onset) which typically provide greater predictive value of health outcomes than sleep duration alone [11].

Despite the ability of accelerometers to measure behaviors across the 24 h spectrum, less is known about metrics that encapsulate the full 24 h that could be derived from accelerometer data. These metrics may identify unique patterns of behavior that could further explain relationships with health outcomes. One such known metric that is ascertained from accelerometry is the rest-activity cycle that can represent the human circadian system. Disruptions in the circadian system consistently show profound and detrimental impacts on health [12] and studies using accelerometry have shown relationships with health-related quality of life and better survival following metastatic colorectal cancer chemotherapy treatments [13, 14].

Recently, with the growth of more “wearable” accelerometers that accommodate larger storage capacities, waterproofing, and more unobtrusive wear locations, long-term monitoring of behaviors (i.e., >1 week) throughout the 24 h spectrum has become more feasible. Indeed, consumer-based accelerometers (e.g., Fitbit, Jawbone) are already achieving long-term population-level data collection of these health behaviors. With the collection of long-term data, it may now be possible to characterize weekly, seasonal, and even annual patterns of behaviors that encapsulate the full 24 h spectrum that extend beyond traditional methods (e.g. accelerometry thresholds, sleep/wake rhythms). Periodicities (i.e., repeating patterns) are observed in many human behaviors and may be derived from various forms of lifelog data [15]. However, such methods have not been applied to long-term monitoring of accelerometry data. Therefore, our primary purpose of the work reported in this paper was to develop a framework for identifying meaningful periodicities (i.e., repeating patterns) from longitudinal wrist-worn accelerometer data. Secondarily, we sought to establish whether these periodicities were independently associated with key cardiometabolic biomarkers and health-related quality of life. We applied five different methods to calculate intensity of the rest-activity cycle and show how each method performed in terms of correlation with biomarkers and health-related quality of life and to see if there was a consistent, or any kind of, pattern across the five methods.

## 2. Materials and Methods

### 2.1. Participants

Participants were drawn from a smartphone-based, multicomponent behavioral intervention targeting changes in sleep, sedentary behavior, and more active behaviors. The target population was US Veterans currently receiving clinical care at a regional Veterans Health Administration (VHA) hospital in the Southwestern United States, aged 35–65 years, measured overweight/obese (BMI ≥25 kg/m^2^), with a fasting glucose of ≥100 mg/dL. Eligibility criteria also included reporting of (a) insufficient physical activity (defined as endorsing activity ranking categories ≤4 on the Stanford Brief Activity Survey [16], which closely aligns with national physical activity guidelines), excessive sitting (defined as ≥8 hours of sitting from the International Physical Activity Questionnaire (IPAQ) [17]), and short sleep duration (<7 hours/night) or mild/moderate sleep complaint (modified version of the Insomnia Severity Index (ISI) [18]). All participants completed telephone screening to determine eligibility. Institutional review boards governing the local VHA hospital and the university to which some of the researchers were affiliated approved all study procedures. All participants provided written informed consent.

### 2.2. Procedures

Participants were initially screened by telephone and this was followed by an in-person visit to confirm eligibility and complete informed consent procedures. At this visit, participants were given a wrist-worn accelerometer for three consecutive weeks. This period constituted the “run-in” period of the behavioral intervention and baseline data collection period. Participants were instructed to wear the monitor continuously during both sleep and wake. Participants were able to remove the accelerometer but were encouraged to wear the monitor as continuously as possible. As part of the run-in period, participants were asked to self-monitor their sleep, sedentary, and active behaviors using a customized smartphone application designed for this purpose. After two weeks, participants were mailed a second accelerometer and asked to return the first accelerometer in a prepaid envelope. At three weeks, participants returned for a second in-person visit where the second accelerometer was returned and all other study measures including questionnaires, blood draws, and clinical measurements were completed. Participants received $25 USD for completing study measures at this visit. Following this visit, participants were randomized to receive active elements of the behavioral intervention. A full description of the intervention is beyond the scope of this investigation and is discussed elsewhere [19], but briefly, participants were randomized into a full-factorial 2 × 2 × 2 screening experiment where smartphone-based interventions targeting sleep, sedentary behavior, and physical activity were delivered for 8 weeks. All participants maintained self-monitoring of their behaviors using the custom application during the intervention phase. Participants also attended two additional visits during the eight weeks to complete study-related assessments and to return/exchange accelerometers to maintain continuous wear. To take advantage of the continuous and longitudinal nature of the data, the full accelerometer data for the run-in and intervention periods were leveraged for this analysis and the effect of the intervention was statistically controlled for in all analyses.

### 2.3. Measures

#### 2.3.1. Lifelog Accelerometry

Movements during sleep and wake were monitored objectively and continuously throughout the study period using the GENEactiv accelerometer (Activinsights, Kimbolton, UK). The GENEActiv is an open source, wave-form wrist-worn accelerometer that is fully waterproof, allowing the monitor to be worn continuously, 24 h a day, without the need to be removed during water activities or be shifted from hip to wrist for daytime and nighttime measurement. Since the GENEactiv provides continuous forms of data recordings for periods of at least 1 month, it can be considered a valid form of lifelogging. Data captured on board the device were initially sampled at 40 hz and summarized to 60 s epochs using a gravity-subtracted sum of vector magnitudes provided through the Activinsights software package [20]. Periods of nonwear were screened for and removed based upon variability in the monitor temperature outputs (i.e., low variability indicates lack of normal fluctuation in temperatures indicated of human wear) and visual inspection. Additional removal occurred for overlapping wear periods that occurred when the monitors were in transit by post.

#### 2.3.2. Cardiometabolic Outcomes

Clinical assessments of waist circumference and blood pressure were taken. Waist circumference was measured at the end of normal expiration at the level of the iliac crest by wrapping a flexible measuring tape snugly around the waist with the tape parallel to the floor. Blood pressure was measured twice (five minutes apart) in the seated position after 10 minutes of rest with a single, regularly calibrated, automated blood pressure machine (Casmed 740). Laboratory-based biomarkers were measured following a >9 h fast. A full lipid profile with total cholesterol, high-density lipoprotein (HDL), and low-density lipoprotein (LDL) as well as high-sensitivity C-reactive protein (hsCRP), triglycerides, plasma glucose, and insulin levels was measured. All assays were processed in the VHA clinical laboratory.

#### 2.3.3. Health-Related Quality of Life

A single “general health” quality of life metric was derived from the RAND-36 measure [21], which is similar to those of the Medical Outcomes Study SF-36 [22].

#### 2.3.4. Study Covariates

Sociodemographic and health behavior/status variables considered as potential confounders induced age, gender, race/ethnicity (Caucasian, African-American, Hispanic, and Asian American), leisure-time physical activity (assessed with a metabolic equivalent score from the walking, moderate, and vigorous leisure activities items from the IPAQ [17]), and insomnia symptoms (assessed with a total score from the ISI [18]). Intervention effects were also adjusted for in all models based upon the 2 × 2 × 2 factorial experiment.

#### 2.3.5. Power Spectral Density (PSD) Estimation

PSD estimation can be used to detect significant periodicity or repeating cycles in any kind of signal, including lifelog data. Our previous work showed detected periodicities in several lifelog datasets using various PSD estimation methods [15]. When it is applied to any form of lifelogging, the periodogram can be used to detect the natural cycles that occur in lifestyle, behavior, and activities. Periodicity can be observed in many natural phenomena, such as circadian rhythms associated with our sleep, for example. Intuitively, we think of our routine daily lives as composed of various forms of recurring events with obvious periodicities around daily, weekly, monthly, seasonal, and annual cycles. In any kind of spectral analysis of a lifelog, we expect to see periodicity around these frequencies. However, without the help of lifelogging devices and the resulting lifelog of data, analyzing the periodicity of human life is not a practical proposition.

*(1) Periodogram*. A periodogram is a visualization of the PSD for a continuous spectrum of frequencies calculated from a stream of data values. Periodogram is widely used to estimate spectrum of both discrete and continuous signals in engineering, astronomy, biology, and physics [23]. When periodograms are applied to lifelogs, they can reveal the cycles which form a natural part of human behavior. Periodograms work best when the lifelog data is sampled at a regular frequency and is continuous, without missing values [24]. Missing data was minimal in this application.

Suppose our complete input data sequence is formalized as *x*(*n*), *n* = 0,1,…, *N* − 1. The normalized Discrete Fourier Transform (DFT) of the sequence is defined as

(1) $$\begin{array}{c} X\left(\;f_{k/N}\;\right)=\frac{1}{\sqrt{N}}\overset{N-1}{\underset{n=0}{\sum }}x\left(\;n\;\right)e^{-j2\pi kn/N}, \end{array}$$

where the subscript *k*/*N* denotes the frequency that each coefficient captures. Suppose that *X* is the DFT of a sequence *x*(*n*). The periodogram *P* is provided by the squared length of each Fourier coefficient:

(2) $$\begin{array}{c} P\left(\;f_{k/N}\;\right)={\left\|\;X\left(\;f_{k/N}\;\right)\;\right\|}^{2}\;k=0,1,\ldots ,\left\lceil \frac{N-1}{2}\right\rceil . \end{array}$$

Notice here that *k* ranges from 0 to (*N* − 1)/2. In order to find the *k* dominant periods, we need to pick the *k* largest values of the periodogram. This works well for short to medium length periods but, for long periods with low frequencies, performance is worse because each value in the periodogram *N* indicates the power at frequency interval [*N*/*k*, *N*/(*k* − 1)] which is too wide to capture large periodicity. Thus, the accuracy of periodicity detection at low frequency will be lower than at higher frequency. For lifelogging, this means there is difficulty in detecting patterns measured in years. Another difficulty when using periodograms is spectrum leakage [25], which causes frequencies that are not integer multiples of the DFT bin width to disperse over the entire spectrum which could result in false alarms being detected in the periodogram. Despite this, the periodogram is still an acceptable way to guarantee the accuracy of detected periods with short to medium frequency.

*(2) Least-Squares Spectral Analysis*. Past work [23] has shown that the Lomb-Scargle periodogram that handles missing data values can be successfully applied to generate periodograms from noncontinuous lifelog data.

Least-squares spectral analysis or LS periodogram is a very different method developed by Lomb and Scargle based on work by Barning and Vanicek to handle continuous data with missing parts [26, 27]. To formalize the problem, suppose we have a data sequence with *N* data points: *X*_*n*_ = *X*_*t*_*n*__, *n* = 0,1,…, *N* − 1. The mean and variance of the data sequence need to be calculated first.

The Lomb-Scargle periodogram has the following expression:

(3) PXω=C∑n=1Nytncos⁡ωtn−τ2∑n=1Ncos2⁡ωtn−τ+∑n=1Nytnsin⁡ωtn−τ2∑n=1Nsin2⁡ωtn−τ,

where *C* could be 1/2 or 1/2*σ* ^2^ and *τ* is defined as

(4) $$\begin{array}{c} \tan\left(\;2\omega \tau \;\right)=\frac{\sum_{n=1}^{N}\sin\left(2\omega t_{n}\right)}{\sum_{n=1}^{N}\cos\left(2\omega t_{n}\right)}. \end{array}$$

*(3) Periodogram of Autocorrelation Function.* In statistics, correlation is basically used to measure how similar two sequences are. This quantitative measurement of similarity of signal 1 and signal 2 can be defined as

(5) $$\begin{array}{c} r_{12}=\frac{1}{N}\overset{N-1}{\underset{n=1}{\sum }}x_{1}\left[\;n\;\right]x_{2}\left[\;n\;\right]. \end{array}$$

A cross-correlation between time shifted sequences can be defined as

(6) $$\begin{array}{c} r_{12}\left(\;k\;\right)=\frac{1}{N}\overset{N-1}{\underset{n=1}{\sum }}x_{1}\left[\;n\;\right]x_{2}\left[\;n+k\;\right]. \end{array}$$

All possible *k*-shifted time series could generate another sequence of numbers only changing with *k*, which is called full cross-correlation. The correlation between a signal and the time shifted version of itself is called an autocorrelation. A lag operator is used to generate the time shifted signal and “0 lag” equals to mean-square signal power. Autocorrelation can be defined as

(7) $$\begin{array}{c} r_{11}\left(\;k\;\right)=\frac{1}{N}\overset{N-1}{\underset{n=1}{\sum }}x_{1}\left[\;n\;\right]x_{1}\left[\;n+k\;\right]. \end{array}$$

We can observe that if the signal is periodic, the normalized autocorrelation is also periodic. Based on this, it is interesting to use the periodogram of the autocorrelation as a PSD estimator. The following equation is used to calculate the periodogram of autocorrelation function:

(8) $$\begin{array}{c} R\left(\;f_{k/N}\;\right)=\frac{1}{N}{\left\|\;\sum_{i=0}^{N-1}r_{11}\left(\;i\;\right)e^{-j2\pi ki/N}\;\right\|}^{2}. \end{array}$$

*Periodicity Strength*. Since 24 h/circadian periodicity is observed and significant in almost all lifelog data generated by human subjects, we would like to use the lifelog to compute the strength of the circadian periodicity for each participant at different points in time. Based on the PSD calculated from the input data, we try to estimate the periodicity strength at given times using different methods and thereafter compare those strengths with markers of cardiometabolic risk and health-related quality of life.

We use the following denotation to explain how we calculate the strength of periodicity.

*ℱ* denotes the DFT of signal *x*(*n*), *n* = 0,1,…, *N* − 1, and *ℱ*′ denotes the inverse transformation. *S* stands for the strength of periodicity. The autocorrelation was calculated using five different approaches, described as follows:

(9) A1k=1k∑n=1N−1xnxn+k,A2k=∑n=1N−1xnxn+k.

Method 1:

(10) S=Pfwhere  f=1day,Pf=1NFxn2.

Method 2:

(11) S=Pfwhere  f=1day,Pf=1NFA1xn2.

Method 3:

(12) S=Pfwhere  f=1day,Pf=1NFA2xn2.

Method 4:

(13) S=max⁡Pf,Pf=1NFxn2.

Method 5:

(14) S=12∑nxn−xn′2,xn′=F′Pfif  f≠1day,  Pf=0,Pf=Fxn.

Method 1 uses power carried by 1/day frequency as the strength of circadian periodicity, namely, the correlation between signal and sinusoid with daily periodicity. Methods 2 and 3 use *𝒜*_1_ and *𝒜*_2_ to calculate autocorrelation, respectively. Using the result of autocorrelation as input to compute periodogram, we thereafter use power of daily periodicity as strength of the circadian periodicity. It should be noted that *𝒜*_1_ is normalized autocorrelation. Method 4 uses the maximum power in the periodogram to represent strength of periodicity, though in this case it is not assured that daily periodicity will carry maximum power all the time. Finally, Method 5 calculates a sinusoid with daily periodicity that is correlated to the data most and then computes root-mean-square error (RMSE) between the signal and the most-fit sinusoid with daily period.

If we consider the informal formulation of spectrum estimation as estimating how the total power is distributed over the frequency, the definition of intensity of periodicity can be thought of as the power corresponding to a certain periodicity or several periodicities. Method 1 comes directly from the definition of power spectral density, which uses DTFT to calculate how power is distributed over frequency directly and here in method 1 we only take the power carries by 24-hour periodicity. Methods 2 and 3 derive from another definition of power spectrum which shows that spectrum can be achieved as the DTFT of the autocorrelation. Method 3 is normally used to calculate autocorrelation in signal processing. The reason we also use Method 2 is because when we lag signal to calculate autocorrelation, the bigger the lag is, the less number of points is involved in the calculation. Method 2 is trying to eliminate this effect by using averaged value. Both of Methods 2 and 3 use power of circadian periodicity as the intensity. Method 4 is using power of frequency with maximum power as intensity. The reason we are using this method is that we are trying to see how the frequency with maximum power would be correlated with biomarkers. In particular, it is interesting to see the result between Methods 1 and 4. Method 5 uses a different way to calculate how the signal is different from the 24-hour periodicity. The rest of the methods use correlation as a metric to quantify the difference while Method 5 uses summed error as the metric to quantify the deviation.

An intensity graph is generated by using a sliding window with selected window and overlapping sizes to visualize the intensity/strength of the periodicity [28]. Within each window, we calculate the strength of the circadian periodicity using Method 1; thus we can see the intensity/strength of periodicity over time.

*Descriptive Analyses and Relationships with Cardiometabolic and Health-Related Quality of Life Outcomes*. We calculated descriptive statistics to represent the sample including means, SDs, frequencies, and percentages. Multiple linear regression analysis was used to identify which periodicity strength metrics were associated with the cardiometabolic and quality of life outcomes. Partial correlation coefficients, after adjustment for age, gender, race/ethnicity, leisure-time physical activity, insomnia symptoms, and intervention assignment, were used to characterize this relationship. Analyses were conducted using SAS Enterprise Guide 6.1 (SAS Institute, Inc.). Inferential testing was conducted at a *P* < 0.05 significant level; however, due to the relatively small sample size and exploratory nature of the study, moderate effect size correlations and *P*'s < 0.10 were also considered. We considered an *r* effect size of >0.25 to be “moderate” in strength [29].

## 3. Results

### 3.1. Participants

Table 1 provides demographic information regarding the final sample of participants. In total, 24 participants were enrolled for this analysis; however, four were excluded due to not presenting for the study measure completion following the initial three weeks of accelerometer wear. The final sample (*N* = 20) were middle-aged, primarily men and Caucasian, inactive, and with moderate levels of insomnia symptoms. Continuous accelerometer wear time varied from 13.9 (minimum) to 102.0 (maximum) days (mean wear: 64.4 ± 26.2 days). Nonwear time was minimal across the 24 h period in the sample (0.03 ± 0.07 percent of days). Of the 24 participants, 15 had complete data (58.8 ± 26.4 days). The remaining nine participants had 73.6 ± 23.1 days of data collection and 9.2% ± 9.0% days of missing data. Overall missing data across the full data collection period was 3.5% ± 7.1%.

### 3.2. Identification of Periodicities

**Figure 1:**
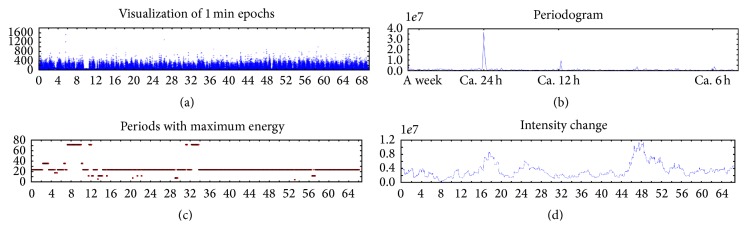
Exemplar data of 24 h behavioral periodicities over 70 consecutive days of wrist-worn accelerometry. 1 min = 1 minute; for more details, see text.

Figure 1 outlines the methodological steps for identifying periodicities and visualizing periodicity strength. Panel (a) provides visualization of the sum of vector magnitudes (1 min epochs) along the *y*-axis and time along the *x*-axis over the course of the monitoring period. Sleep and wake periods are evident visually from these data. Panel (b) displays a periodogram calculated from 1 m epochs. The *x*-axis is frequency and *y*-axis is energy of the frequency, namely, how strong the corresponding frequency is. In Figure 1, we observe strong circadian periodicity followed by a 12 h periodicity which is the harmonic of the circadian. No within-day or weekly patterns were observed. Panel (c) plots time (*x*-axis) by the strongest periodicity observed over the 3-day time lagged window. *y*-axis of Panel (c) is the frequency that carries maximum power within a window. In this example, the 24 h periodicity held consistently for the majority of 3-day windows with small breaks at the beginning of the monitoring period. Panel (d) describes the strength of the periodicity using Method 1 (*y*-axis) over time (*x*-axis). The strength/intensity of the 24 h circadian periodicity changes throughout the lifelogged observation period, showing, for example, a weaker period of regular circadian cycle from day 0 to day 14 and again from day 32 to day 44.

### 3.3. Periodicity Strength Metrics

Table 2 presents descriptive statistics and intercorrelations among the five methods for calculating periodicity. Methods 1–4 displayed very high correlations among methods. In particular, Method 1 was strongly correlated with Method 4 and Method 2 was strongly correlated with Method 3. Method 5 was not strongly correlated (*r*'s < 0.40) with any of the other methods. Normalized versions of these metrics were calculated and similar pattern of results was observed (not pictured).

### 3.4. Associations with Cardiometabolic and Quality of Life Outcomes

Table 3 presents descriptive data for cardiometabolic and quality of life outcomes and partial correlation coefficients for each of the periodicity strength metrics and cardiometabolic and quality of life outcomes. The profile of participants' descriptive data suggests that the sample was at moderate to high risk for cardiometabolic risk diseases. The strongest and most consistent correlations were observed between the periodicity strength metrics and LDL-cholesterol and triglycerides outcomes. Consistent—yet only moderate in strength—relationships were observed for hs-CRP and health-related quality of life. HDL-cholesterol, plasma glucose, and insulin were not consistently associated with the periodicity strength metrics. As expected (due to high intercorrelations), Methods 1–4 displayed very similar pattern of results. In contrast to Methods 1–4, Method 5 displayed a moderately strong relationship with systolic BP and HDL-cholesterol and no relationship with hs-CRP or triglycerides.

## 4. Discussion

The purpose of this study was to develop a framework for identifying periodicities (i.e., repeating patterns) from longitudinal wrist-worn accelerometer data and to establish whether these periodicities were independently associated with key markers of cardiometabolic health and health-related quality of life. The resultant periodograms demonstrated a consistent 24 h pattern representing a typical rest-activity cycle; however, the strength of this 24 h rest-activity pattern varied within and between individuals. Using varying methods of quantifying periodicity strength, we found preliminary evidence that the strength of the rest-activity cycle was associated with key cardiometabolic risk biomarkers and health-related quality of life independent of self-rated physical activity and insomnia symptoms.

Despite different methodologies in characterizing the rest-activity cycle and health outcomes, this study is consistent with other studies. Mormont et al. [13] examined the rest-activity cycle in metastatic colorectal cancer patients using an autocorrelation coefficient at 24 h and a dichotomy index that compared activity in bed and out of bed. These metrics were positively correlated with improved quality of life, response to treatment, and survival. In a follow-up to this study, Innominato et al. [14] further clarified the importance of the rest-activity cycle, as measured via accelerometry, by demonstrating the stronger correlations observed between the rest-activity cycle metrics compared to mean counts of physical activity for health-related quality of life and survival outcomes in metastatic colorectal cancer patients. Our study extends these findings in some important ways. First, these studies sampled behavior over 3-4 consecutive days. Therefore, our investigation substantially lengthens the monitoring period and therefore provides a clearer picture of habitual 24 h rest-activity cycles. Second, we have explored these relationships and found associations with a broader set of health outcomes in a group at elevated cardiometabolic risk. Finally, our framework for the development of periodograms and metrics to characterize periodicity strength represents a more sophisticated and nuanced approach that may provide a more precise determination of the 24 h rest-activity cycle.

One of the most interesting findings from the current investigation was the differences and similarities in correlation of the various periodicity strength metrics and health outcomes. Methods 1–4 yielded very similar results due to high intercorrelations among these related methods for quantifying periodicity strength. Methods 1–4 computed correlation as the sum of products (*s∗r*) over all points, *s*_*i*_, in the pattern against corresponding points *r*_*i*_, in the original signal. This tells us how close the shape is between the original signal and the detected pattern. These metrics were consistently and strongly correlated with cardiovascular physiology outcomes such as LDL-cholesterol, triglycerides, and inflammation (hs-CRP). In contrast, Method 5 produced a different profile of correlation with health outcomes. Method 5 computed correlation as the root-mean-square error of the difference (*s* − *r*) between corresponding points in the original data and the pattern. This tells us the sum of absolute differences between the pattern and the original signal. This metric was associated with HDL-cholesterol and systolic blood pressure, while Methods 1–4 were not. While it is not directly clear why these unique correlates were identified for the various periodicity strength metrics, it does suggest that nuances in the rest-activity cycle may uniquely contribute to cardiometabolic disease risk.

### 4.1. Strengths and Limitations

An important strength of this study was the long-term, longitudinal nature of the collection of accelerometry data. Typically, accelerometer data are collected for seven or fewer consecutive days. These analyses demonstrate a novel methodology for harnessing longitudinal accelerometry data with demonstrated additional explanatory power for health outcomes beyond what has typically been reported in reports of accelerometry data and health outcomes. An additional strength was the minimal missing accelerometer data. While the methods employed here were relatively robust to missing data, the trivial missing data demonstrates the feasibility of collecting long-term monitoring data. A final strength of this study was the reliance on a completely open-source, raw data collection methodology with no proprietary algorithms. An important limitation of this preliminary study was the relatively small sample size and limited duration of the monitoring period. While there was substantial within- and between-person variability in periodicity strength observed, a larger sample may have yielded stronger and more definitive patterns in the rest-activity cycle. Relatedly, the sample was exclusively those with elevated cardiometabolic risk and the results may not generalize to a healthy population. Furthermore, while the length of the monitoring was indeed longer than typically what is reported, longer monitoring periods may have yielded more interesting month, seasonal, or annual patterns of data as have been observed in other forms of lifelog data [15]. Additionally, while the periodicity strength metrics were calculated based on longitudinal data, the cardiometabolic and health-related quality of life metrics were measured concurrently, and therefore the relationships reported represent cross-sectional associations. Finally, these data were collected in the context of a behavioral intervention. While the effect of this intervention was statistically adjusted for, residual confounding may exist.

### 4.2. Future Directions

Logical next steps for this work are threefold. First, replication of these methods in larger and more diverse samples is warranted. This may include the use of existing cohorts where raw data collection protocols of 24 h accelerometry are in place (even protocols that only include seven days of wear) with health-related outcomes measured in a cross-sectional or longitudinal fashion (e.g., US National Nutrition and Health Examination Survey, UK Biobank). Second, if these metrics are further shown to be related to health outcomes, it becomes critical to understand whether these metrics may be amenable to behavioral intervention and by what means this may be possible. It is not known whether this metric may be more sensitive to changes in sleep, sedentary behavior, physical activity, some combination of these behaviors, or some alternative strategy not currently being considered. Given this metric's independence from physical activity and sleep, it may require novel intervention strategies. Further clarification is needed for why these metrics were associated with certain biomarkers and not others, as well as why there was such variability in the strength of these associations with various biomarkers. Carefully laboratory-based studies that seek to experimentally manipulate the rest-activity cycle (and consequently change periodicity strength) may be useful in understanding the physiological mechanisms underlying these relationships. Finally, because of the cross-sectional nature of the current study, causality cannot be established, and therefore it is imperative that these relationships be followed longitudinally where periodicity strength is experimentally manipulated in a manner to invoke changes in cardiometabolic risk biomarkers. This would provide greater clarity regarding the overall direction of the mechanistic effects.

## 5. Conclusion

The use of periodograms and periodicity strength represents a novel methodology for understanding long-term monitoring of 24 h accelerometry data. This analytical framework can be used with minimally processed accelerometer data and, in this sample, demonstrated moderate to strong independent associations with key cardiometabolic and health-related quality of life outcomes. This framework and preliminary work may be useful as long-term monitoring of accelerometer data across the 24 h becomes more commonplace in epidemiological and intervention research.

## Figures and Tables

**Table 1:** Participant demographics (*N* = 20).

Age, M ± SD	49.7 ± 9.1
Men, *N* (%)	17 (85.0)
Race/ethnicity, *N* (%)	
Caucasian	14 (70.0)
African-American	3 (15.0)
Hispanic	2 (10.0)
Asian American	1 (5.0)
Leisure-time physical activity (MET-min/week), M ± SD	878.6 ± 1680.9
Insomnia symptoms (ISI), M ± SD	14.8 ± 6.4

ISI = Insomnia Severity Index (range: 0–28).

**Table 2:** Means, standard deviations, and Pearson correlations among five periodicity strength metrics (*N* = 20).

	Method 1	Method 2	Method 3	Method 4	Method 5
Mean	0.20	0.10	0.10	0.23	0.45
SD	0.24	0.22	0.21	0.24	0.15

Method 1					
Method 2	0.93				
Method 3	0.93	0.99			
Method 4	0.98	0.90	0.92		
Method 5	0.29	0.14	0.19	0.38	

**Table 3:** Partial correlation coefficients, between cardiometabolic biomarkers and health-related quality of life indices, and periodicity strength metrics (*N* = 20).

	M ± SD	Periodicity strength metrics				
		Method 1	Method 2	Method 3	Method 4	Method 5
Waist circumference, in	66.82 ± 35.10	0.28	0.27	0.25	0.30	‡
Systolic BP, mm Hg	138.6 ± 17.13	‡	‡	‡	‡	0.57^*∗*^
Diastolic BP, mm Hg	89 ± 16.32	‡	‡	‡	‡	‡
Total cholesterol, mg/dL	177.4 ± 50.51	0.52^†^	0.68^*∗∗*^	0.57^*∗*^	0.46^†^	0.47^†^
HDL cholesterol, mg/dL	33.9 ± 11.76	‡	‡	‡	‡	0.51^†^
LDL cholesterol, mg/dL	109.7 ± 37.64	0.45^†^	0.57^*∗*^	0.46^†^	0.40	0.42
hs-CRP, mg/dL	7.76 ± 5.60	0.47^†^	0.38	0.30	0.53^†^	‡
Triglycerides, mg/dL	168.7 ± 74.06	0.77^*∗∗*^	0.86^*∗∗∗*^	0.81^*∗∗∗*^	0.75^*∗∗*^	‡
Plasma glucose, mg/dL	117.2 ± 50.69	‡	‡	‡	‡	‡
Insulin, pmol/L	44.58 ± 73.01	‡	‡	‡	‡	‡
Health-related quality of life	47.25 ± 13.03	0.37	0.54^*∗*^	0.55^*∗*^	0.37	0.52^†^

^*∗∗∗*^ *P* < 0.001; ^*∗∗*^ *P* < 0.01; ^*∗*^ *P* < 0.05; ^†^ *P* < 0.10; ^‡^ *r* < 0.25 and *P* > 0.0.

All models are adjusted for age, gender, race/ethnicity, leisure-time physical activity, insomnia symptoms, and intervention assignment.
